# Healthcare providers perspectives on compassion training: a grounded theory study

**DOI:** 10.1186/s12909-020-02164-8

**Published:** 2020-08-05

**Authors:** Shane Sinclair, Thomas F. Hack, Susan McClement, Shelley Raffin-Bouchal, Harvey Max Chochinov, Neil A. Hagen

**Affiliations:** 1 Faculty of Nursing, University of Calgary, 2500 University Drive NW, Calgary, Alberta T2N 1N4 Canada; 2 Division of Palliative Medicine, Department of Oncology, Cumming School of Medicine, University of Calgary, 2500 University Drive NW, Calgary, Alberta T2N 1N4 Canada; 3 Compassion Research Lab, University of Calgary,; 4 Research Institute in Oncology and Hematology, CancerCare Manitoba, Winnipeg, Manitoba Canada; 5 College of Nursing, Rady Faculty of Health Sciences, University of Manitoba, 89 Curry Place, Winnipeg, Manitoba R3T 2N2 Canada; 6 Psychosocial Oncology & Cancer Nursing Research, I.H. Asper Clinical Research Institute, 369 Taché Ave, Winnipeg, R2H 2A6 Manitoba Canada; 7 Department of Psychiatry, University of Manitoba, 771 Bannatyne Avenue, Winnipeg, Manitoba R3E 3N4 Canada; 8 Departments of Clinical Neurosciences and Medicine, Cumming School of Medicine, University of Calgary, 2500 University Drive NW, Calgary, Alberta T2N 1N4 Canada

**Keywords:** Compassion, Grounded theory, Healthcare training, Compassion training, Compassionate care, Model

## Abstract

**Background:**

There is little concrete guidance on how to train current and future healthcare providers (HCPs) in the core competency of compassion. This study was undertaken using Straussian grounded theory to address the question: “What are healthcare providers’ perspectives on training current and future HCPs in compassion?”

**Methods:**

Fifty-seven HCPs working in palliative care participated in this study, beginning with focus groups with frontline HCPs (*n* = 35), followed by one-on-one interviews with HCPs who were considered by their peers to be skilled in providing compassion (*n* = 15, three of whom also participated in the initial focus groups), and end of study focus groups with study participants (*n* = 5) and knowledge users (*n* = 10).

**Results:**

Study participants largely agreed that compassionate behaviours can be taught, and these behaviours are distinct from the emotional response of compassion. They noted that while learners can develop greater compassion through training, their ability to do so varies depending on the innate qualities they possess prior to training. Participants identified three facets of an effective compassion training program: self-awareness, experiential learning and effective and affective communication skills. Participants also noted that healthcare faculties, facilities and organizations play an important role in creating compassionate practice settings and sustaining HCPs in their delivery of compassion.

**Conclusions:**

Providing compassion has become a core expectation of healthcare and a hallmark of quality palliative care. This study provides guidance on the importance, core components and teaching methods of compassion training from the perspectives of those who aim to provide it—Healthcare Providers—serving as a foundation for future evidence based educational interventions.

## Background

The ability to provide compassion has become an expected core competency for healthcare providers (HCPs), mandating the need to understand, develop and evaluate training opportunities for the provision of compassion [1–4]. While the topic and feasibility of how to train HCPs to provide compassion is contentious, patients feel it is not only possible, but necessary [5]. Despite this endorsement, there are challenges to developing compassion training programs—including a lack of conceptual clarity, a lack of clinical research measuring the impact on patient care, and limited training on skills and behaviours associated with compassion. Recent research has shown that compassion is a dynamic, individualized and complex construct that stems from innate virtues of the healthcare provider. It is conveyed through relational communication, an in-depth understanding of the person and a range of clinical behaviours that are not conducive to a strict didactic approach or rote learning [6, 7].

**Fig. 1:**
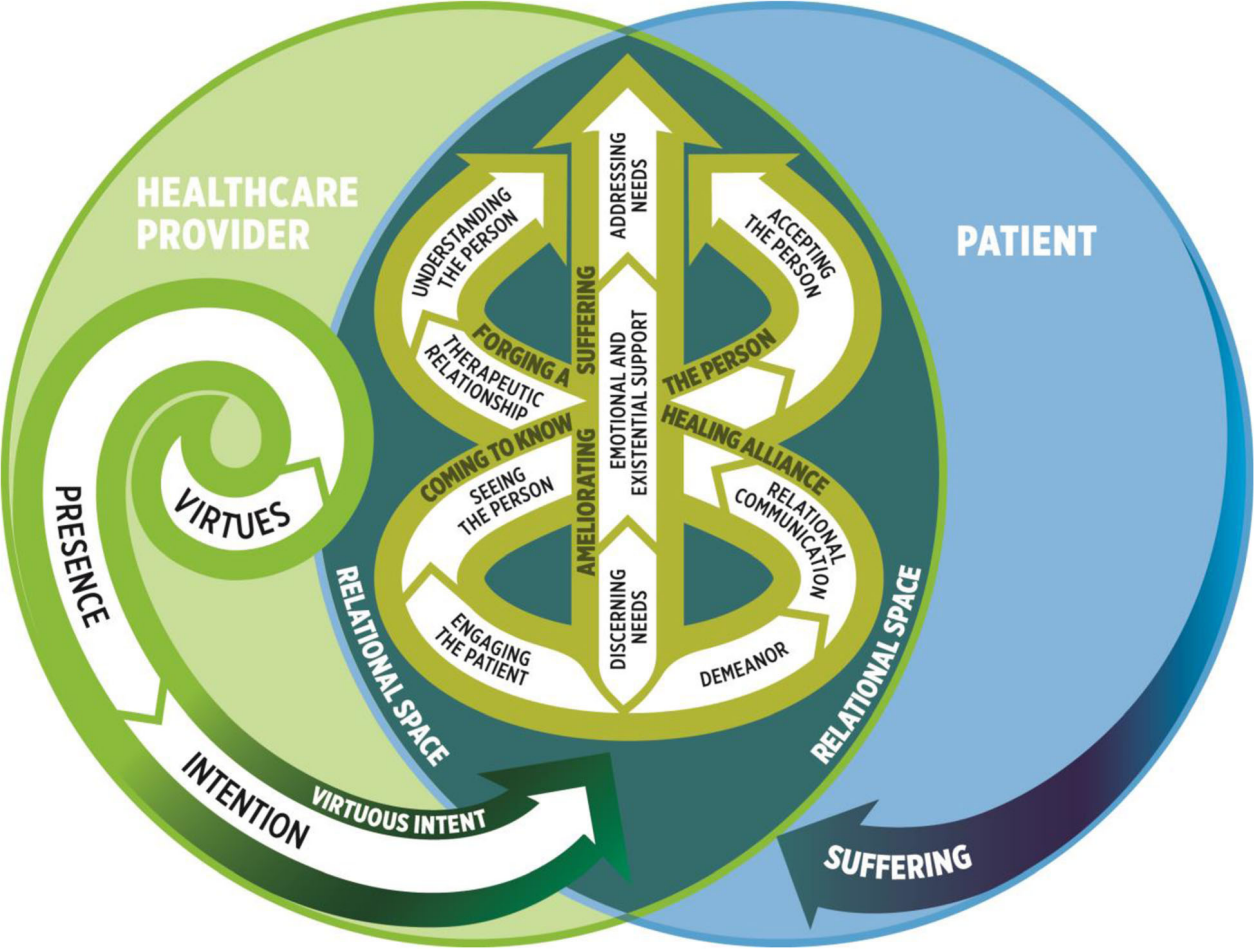
Healthcare Provider Compassion Model

Compassion has been simply defined as a response to the suffering of others that motivates the desire to alleviate it [8]; however, the provision of compassion is complex, involving a broad range of qualities, skills and behaviours which together focus on the alleviation of suffering [1]. These include: acting with warmth and understanding [1, 6, 7], providing personalized care [1, 6, 7, 9], acting toward a patient the way you would want them to act toward you [1], providing encouragement [1, 6, 7, 9], communicating effectively [9], and acknowledging a patient’s emotional issues [1, 6, 7, 10]. Importantly, compassion requires HCPs to engage suffering on both a personal and professional level, using interpersonal skills to care for patients and intrapersonal skills to care for themselves as caregivers [6, 7]. While patients and HCPs agree that compassion involves a multitude of skills and behaviours, they need to be coalesced with virtues such as love, genuineness, acceptance and kindness to be considered compassionate, suggesting that the cultivation of virtues is a key, albeit challenging, component of compassion training [5, 6, 9]. The complexity and multiple domains of compassion are embedded within the following definition of compassion, which will be used herein--a *virtuous and intentional response to know a person, to discern their needs, and ameliorate their suffering, through relational understanding and action* [7]. The core components of compassion within this definition generated the healthcare provider compassion model (Fig. 1).

The benefits of compassion include: improved healthcare provider-patient relationships; higher patient satisfaction; reduced patient anxiety; higher pain tolerance; and an improved stress response [3, 10, 11]. Notably, patients and family members who perceive a lack of compassion in their healthcare experience may have more adverse medical events, poorer symptom management, and are more likely to lodge complaints and sue for malpractice [12–17]. Publications characterizing the impact of compassion on HCPs have largely focused on the potential negative outcomes under the rubric of compassion fatigue, a concept that has recently been questioned by researchers and HCPs alike, while noting the beneficial aspects of compassion [3, 18–21]. High profile healthcare failures have been attributed to deficiencies of compassion within the healthcare system, and have resulted in calls to enhance competency training in compassionate healthcare delivery [13, 22–25]. The findings and recommendations of these reports have highlighted the necessity for compassion training of future HCPs, with over half (58%) of current HCPs in the United Kingdom identifying that students need additional training in order to provide compassion in practice [1]. There is emerging evidence that attitudes and qualities associated with compassion can be nurtured, and that compassionate behaviors can be taught through formal instruction [5, 26, 27]. However, there is no consensus in the literature as to the best approaches for training HCPs in compassion and there is a dearth of evidence-based compassion training programs to equip healthcare students and providers in the process.

The objective of this study was to address the research question “What are healthcare providers’ perspectives on training current and future HCPs in compassion?” in order to generate evidence to inform the development of learner-informed, clinically relevant, evidence-based training interventions to equip current and future HCPs in providing compassion.

## Methods

### Study population

This study was conducted using Straussian grounded theory to directly understand healthcare providers’ perspectives on compassion [7, 28–31], including their perspectives on compassion training, which is the focus of the current article. Grounded theory studies focus on theory development, with data collection and analysis occurring concurrently, allowing for subsequent data collection to be informed by the emerging theory. Participants in this study were part of a larger study that investigated healthcare providers’ broader perspectives and experiences of providing compassion. Generating a healthcare provider model of compassion (Fig. 1) [7]. In grounded theory studies, facets of the larger theory are often analyzed and reported independently, which led the research team to prospectively develop specific guiding questions related to the topic of compassion training at the design stage, with the results being reported exclusively herein [6, 32].

The participants in this study were palliative care providers (*n* = 57) working in a variety of settings (palliative care unit, hospice, homecare, hospital based palliative consult service) in urban and rural Alberta, Canada. Individuals were eligible to participate if they: 1) were ≥ 18 years of age; 2) were able to read and speak English; 3) had worked in palliative care for at least 6 months; and 4) were able to provide written informed consent. In the first stage, frontline palliative care providers (*n* = 35) participated in one of seven focus groups in order to elicit the perspectives of individuals who are keenly aware of the pragmatic skills, clinical challenges and requisite knowledge base that is necessary when providing compassion within the demands of a healthcare system. For Stage 2, interview participants (*n* = 15, three of whom were also focus group participants) were nominated by focus group participants in response to the following question on the study demographic form “Who among your interdisciplinary palliative care peers do you feel possesses great skill in providing compassion?” Stage 3 focus groups with study participants (*n* = 5) and knowledge users (*n* = 10) consisting of clinical educators, faculty, administrators and clinical leaders were conducted to verify the categories and themes generated in stages 1 and 2 in order to achieve data saturation. Stage 3 participants were also asked additional knowledge translation questions in order to glean insight on the implementation of compassion training into university curricula and continuing healthcare education. While we anticipated needing a large sample to comprehensively address the study objectives, sample sizes are not predefined in qualitative research but rather determined when data saturation is reached, which in this study occurred after conducting focus groups and interviews with 57 participants.

### Data collection and analysis

Data were collected over a 12-month period using interview guides based on our review of the literature and based on previous experience conducting qualitative research on the topic of compassion in healthcare (Table 1) [2, 6, 7]. After receiving approval from the University of Calgary Conjoint Health Research Ethics Board (#REB 15–1999), study participants were recruited via study posters and in-services, through convenience, snowball and theoretical sampling. Stage 2 interview participants were contacted in rank order based on the number of nominations they received from Stage 1 participants. Stage 2 participants were informed about their nomination and the study via a work email asking them if they would be willing to participate in a semi-structured interview.

**Table 1:** Guiding Questions Utilized in Focus Group and Semi-Structured Interviews that Pertain to Compassion Training

**Stage 1 Focus Group Guiding Questions**	
1 Based on your professional and personal experience, what does compassion mean to you?	
2 Can you give me an example of when you felt you provided or witnessed care that was compassionate? [What do you feel were the key aspects of these interactions?]	
3 What do you feel are the major influencers of compassion in your practice?	
4 What do you feel inhibits your ability to provide compassion?	
5 Do you think patients and/or family members influence the provision of compassion? [How or how not?], [If yes, what characteristics of patients and/or families, do you feel facilitate or inhibit compassion?]	
6 What advice would you give other healthcare providers on providing compassion?	
7 Do you think we can train people to be compassionate? [If so, how]?	
8 Based on your experience what role, if any, do you feel compassion has in alleviating end of life distress? [What happens when compassion is lacking?]	
9 What impact does providing compassion have on your personally and professionally?	
10 Is there anything related to compassion that we have not talked about today that you think is important or were hoping to talk about?	
**Stage 2 Interview Guiding Questions**	
1 You have been identified by your peers as possessing great skill in providing compassion. What do you feel might be some of the reasons for this recognition? [Why do you think others identify you as a compassionate healthcare provider]?	
2 In your own terms, how would you define compassion? [What does compassion mean to you?]	
3 How did you become a compassionate caregiver? [What beliefs, situations, individuals and/or life experiences in your life and practice do you feel have informed your understanding and provision of compassion? Have you always been that way? [Were you always like that? How did you learn it? Can it be learned?]	
4 If you reflect back on your current position, can you walk me through the best example of when you provided compassion? Please guide me through the process of this encounter in a sequential fashion, highlighting the key components of this interaction from the initial approach to the consequences of this interaction?]	
5 Based on your professional and personal experiences, what shapes your compassion?	
6 If you were responsible for training students in compassion, how would you go about it? [What would you teach them?]	
7 Is there anything that gets in the way of your ability to provide compassion?	
8 How do patients and/or families influence your ability to provide compassion? [What characteristics of patients and/or families, do you feel facilitate or inhibit compassion?]	
9 A number of participants have identified the healthcare system as being a significant factor in delivering compassion? From your perspective, how does/can the healthcare system facilitate or inhibit compassion?	
10 In light of the things you’ve just identified as facilitators and barriers, what suggestions would you have for enhancing compassion at a systems level? [Where and what would you focus your efforts on in order to enhance compassion at a systems level]?	
11 From what you’ve told me so far, it sounds like compassion is important. So what happens [to patients, families or HCPs] when compassion is lacking?	
12 What impact does providing compassion have on you personally and professionally?	
13 Our focus group participants, previous studies and review of the literature have reported how critical and fundamental compassion is to providing quality patient care, but we also know that compassion varies. So given all that we know about the importance of compassion, why aren’t healthcare providers more compassionate?	
14 Before we end, given all we’ve talked about, I just want to revisit one of the first questions I asked, which is how do you personally define compassion? [In light of our discussion, what does compassion mean to you?]	
15 Is there anything related to compassion that we have not talked about today that you think is important or were hoping to talk about?	
**Stage 3 Focus Group Questions**	
1 Does the healthcare provider model of compassion make sense to you? [Does it resonate with you]? [Why or Why not]?	
2 Do you feel there is anything missing from the model?	
3 How do you feel this model might be relevant to you and your work?	
4 How do you suggest the model might be integrated into healthcare practice and education?	
5 Is there anything related to the model that we have not talked about today that you think is important or were hoping to talk about	

All participants volunteered (non-remunerated) for this study and provided written informed consent. Interviews and focus groups were conducted by a seasoned qualitative interviewer in a private room in the participant’s place of work at a time that was mutually convenient. Interviews and focus groups were audio recorded with the interviewer recording non-verbal content, such as group dynamics and emotions, in written field notes. Audio files were transcribed verbatim by a professional transcriptionist, with each transcript being cross-verified by the qualitative interviewer after comparing the transcript with the original audio recording.

Focus group and individual interviews ranged from 1 to 1.5 h in duration. Demographic information was collected for each participant (Table 2). Data on the topic of compassion training were elicited through targeted questions pertaining to compassion training (Table 1**,** Stage 1 question #6, 7; Stage 2 question #3, 6; Stage 3 question #4) and other interview guiding questions within the primary study focused on HCPs perspectives and experiences of compassion, which indirectly elicited responses about compassion training (e.g. identifying a lack of education as a barrier to compassion). In developing the interview guide, we intentionally used the term ‘training’ rather than ‘education’ as patient study participants in our previous qualitative research consistently interpreted the term ‘education” as didactic learning or formal lectures, and based on this understanding felt that educating healthcare providers about compassion was not feasible [5]. While the interview guide provided a framework for the interviews and focus groups, the qualitative interviewer was permitted to interject additional probes based on the participants’ responses.

**Table 2:** Participants’ characteristics

Mean Age (Years)	48.6
Men	14 (8)
Women	86 (49)
Mean number of years in palliative care (range)	11.8
Employment Status*	
Full-time	57.8 (33)
Part-time	33.3 (19)
Casual	7.0 (4)
Profession	
Registered Nurse	45.6 (26)
Physicians	22.8 (13)
Healthcare Aide	7.0 (4)
Spiritual Care Specialist	5.2 (3)
Unit Clerk	3.5 (2)
Occupational Therapist	3.5 (2)
Licensed Practical Nurse	3.5 (2)
Housekeeper	1.7 (1)
Social Worker	1.7 (1)
Psychologist	1.7 (1)
Respiratory Therapist	1.7 (1)
Physiotherapist	1.7 (1)
Care Setting**	
Home Care	29.8 (17)
Hospice	26.3 (15)
Hospital Dedicated Palliative Care Unit	21.0 (12)
Hospital Palliative Care Consult Service	14.0 (8)
Palliative Care Administrator	7.0 (4)
Outpatient Oncology Palliative Care Consult Service	5.2 (3)
Rural Palliative Care Consult Service	5.2 (3)
Other	1.7 (1)
Religious Affiliation*	
Christian	52.6 (30)
Buddhist	7.0 (4)
Jewish	3.5 (2)
Muslim	1.7 (1)
Hindu	1.7 (1)
None	31.5 (18)
Religious and Spiritual Status*	
Spiritual and Religious	33.3 (19)
Spiritual but not Religious	56.1 (32)
None	8.7 (5)

*The total for these categories is less than 100% due to nonresponse by participants

**The total for these categories is more than 100% due to some participants working in multiple care settings

Data analysis occurred through the open, axial and selective coding stages of Straussian grounded theory which are reported in detail elsewhere [7, 30]. The analysis team (SS, TH, SM, SRB) have extensive qualitative research experience and expertise, with three members of the team teaching qualitative research courses at their respective universities. In the open coding stage, each member of the team independently read the transcripts, line-by-line, to identify and organize emergent patterns using substantive codes and participants’ actual words. The research team then met and coded each transcript collectively, comparing and contrasting their individual codes until consensus was reached. In the axial coding stage, consensus codes were compared, collapsed and clustered into initial themes and categories creating a coding schema, serving as a repository for the coding of subsequent interviews with additional codes being added as necessary. Finally, selective coding was performed to develop theoretical constructs from the data that identified relationships between categories, validated established relationships and refined categories as required.

## Results

Based on the focus group and interviewee responses, three categories and associated themes were identified that pertained to compassion training within healthcare **(**Table 3**)**. Verbatim quotes from the focus group and interview responses were selected to illustrate each of the categories and themes based on their overall representativeness or to reflect differences of opinions between participants in the response categories.

**Table 3:** Overarching Categories and Themes Identified that Pertain to Training Palliative Care Providers to Provide Compassionate Care

Category	Themes
The feasibility and necessity of compassion training	Teachable Moments: Teach the behaviours and cultivate the qualities The Learner: the role of innate qualities and experience Compassion competency: An educational priority and practice requirement
Training techniques and practices for developing compassion competency	Self awareness Experiential learning Effective and affective communication skills
Sustaining Practices: Self Care and Communities of Compassion	Self-care Communities of Compassion: The need and responsibility of practice settings and healthcare organizations in sustaining compassion training

### Category: the feasibility and necessity of compassion training

While HCPs recognized that compassion training was a challenge, they felt that it was feasible and could be achieved through a variety of teaching techniques, experiential learning opportunities and a learner-centred approach. Three themes were generated from these data: Teachable Moments: Teach the Behaviours and Cultivate the Qualities; The Learner: The Role of Innate Qualities and Experience; and Compassion Competency: An Educational Priority and Practice Requirement.

#### Theme: teachable moments: teach the Behaviours and cultivate the qualities

Participants unequivocally felt that compassionate behaviours (e.g., communication skills, clinical behaviours) could be taught. There was less consensus related to the feasibility of training the emotional and attitudinal domains of compassion. Most participants, however, asserted that the innate facets of compassion could be nurtured through self-awareness, contemplative practices or by practicing compassionate behaviours towards others.

*I think some of those personal characteristics are innate and are traits that you’re born with… But I think there also is an element of education… the more people practice and learn strategies and learn phrases and learn techniques I think it can be improved upon...(Interview Participant 7).*

*I do think compassion’s more than that and so I think you can train the behavioral aspects of it but the emotional and attitude components I’m not sure are so easy (Focus Group Participant 18).*

#### Theme: the learner: the role of innate qualities and experience

A second theme that participants identified in compassion training focused on developing aptitude for the emotional and innate components of compassion. Integral to this aptitude was learners’ pre-existing emotional capacity and past experience with compassion or its antecedent--suffering. Developing an awareness of the suffering of another, being willing to engage the suffering of another, and recognizing suffering as a key facet of the shared humanity between themselves and their patients, were considered essential basic building blocks of compassion in HCPs.

*You have to have some experience with compassion or with that deep sense of trust and understanding somewhere in your life in order to be able to provide that to someone else (Focus Group Participant 4).*

*I don’t know if a person who hasn’t experienced a reason to find compassion or to have compassion be given to them would come by it as innately as other people who have experienced those things (Focus Group Participant 5).*

*I mean a lot of stuff that you do in terms of being able to care and look after people is not something I ever learned in a textbook (Focus Group Participant 2).*

#### Theme: compassion competency: an educational priority and practice requirement

Despite inherent challenges, the majority of participants felt that compassion training needed to be formally embedded within healthcare curricula and be considered a practice competency. Ironically, all participants indicated that the topic was limited in their own healthcare training. While substantive content on the topic of compassion was largely absent, participants felt that in addition to dedicated training on the topic, there were ample opportunities to integrate compassion alongside existing curricula and clinical education opportunities.

*So you know, speaking professionally, honestly, you know there are things that teach compassion, but really, there’s not enough. There just isn’t enough. As a nurse I don’t think in school I learnt enough of that (Interview Participant 2).*

*I think that there is certain things you can teach to help enable compassion to kind of grow and one of those things that I found was actually in nursing school they really harped on it but it makes sense the determinants of health and how all of that comes together to, all these different factors, some of which a person has no control over, such as early childhood education before they’re 2 years old, where they’re born, things like that all come together to bring that person to the situation they’re in. And when you understand some of those factors and that they are out of people’s control I think you can develop a greater compassion just by knowing some of those things (Focus Group Participant 17).*

### Category: training techniques and practices for developing compassion competency

Participants identified several learning techniques or training methods they had personally found effective, suggesting these might be helpful in developing compassion aptitude in others. Within this category, three themes emerged from the data: Self-Awareness; Experiential Learning; and Effective and Affective Communication Skills.

#### Theme: self-awareness

Participants emphasized the importance of developing self-awareness in compassion training and the cultivating virtues or noble qualities that serve as antecedents and markers of compassion within a clinical encounter. In relation to compassion, participants emphasized the necessity of having learners reflect on their own understanding and experiences of compassion and suffering in addition to developing an awareness of their virtues, including, but not limited to, authenticity, vulnerability, honesty, kindness, and love.

*What you bring as a person to that encounter is vitally important and you better know what that is because it will affect how you interact with your patients if you’re not aware of it (Interview Participant 5).*

*I’d also want to do some real inquiry around what their [trainees] conceptualization is of compassion and then talk about what it is and what it isn’t… I’d be curious for them to develop their own awareness of what compassion would look like and how they would tend to it for themselves (Interview Participant 15).*

#### Theme: experiential learning

While participants acknowledged that training future and current HCPs in compassion was likely not easy, most recognized that experiential learning opportunities, rather than didactic learning, were the most effective techniques. This largely seemed to be informed by reflecting on how the participants personally cultivated compassion in their own healthcare training and practice. In particular, participants identified mentors who expressed compassion to their patients as powerful teaching moments, along with personal experiences of being the recipient of compassion.

*Can it be learned? Yes. But I think that no matter how many times you sit in a classroom or how many times someone talks to you about it, or even how many times you witnessed something, until that has an emotional impact on you, you aren’t fully going to grasp what it means to be compassionate (Focus Group Participant 5).*

*I think having, watching people who are experienced and just observing their approach to it as well ‘cause I think people when they first go in they are not sure what to do, right and so probably that internal discomfort makes them more self-conscious and not sure how to approach things, right. And so I think that having someone more experienced to observe and to talk things through afterwards, kind of debriefing, is very helpful (Focus Group Participant 18).*

In addition to preceptors and clinical mentors, patient narratives and role playing that involved learners assuming the role of the patient were important sources of experiential learning. Developing a reflective practice, whereby learners personally reflected on each patient visit or debriefed with a preceptor or clinical mentor on the compassion touch points of a visit, were also identified as experiential learning techniques.

*Either role play or video, but I think video at least as a start to hear from the patient’s perspective… So I think hearing it from the patient’s voice I think is critical in teaching to hear you know, how do patients perceive you (Interview Participant 7).*

*That would be a great thing to do in the course if they actually have to go in and be a patient (Interview Participant 8).*

*I think it’s a reflective thing too. It’s kind of understanding the ways that your actions impacted others and then circling back to so knowing that now, what could I have done differently or what could we have done differently, right? So those feedback loops (Interview Participant 14).*

#### Theme: effective and affective communication skills

The third theme within this category was training related to effective and affective communication skills. Participants emphasized the importance of traditional communication skills such as building rapport, active listening and presenting clinical information in an understandable manner. Beyond this, compassionate communication required the development of more affective skills, such as engaging patients in a sensitive manner, and addressing emotional and existential distress. Communicating support, acceptance and understanding the patient as a person were also central skills identified by participants, as well as routinely eliciting patient feedback about whether they felt they were being treated with compassion.

*And you have to ask the person who’s on the other end of the intended compassion right, whether they feel that compassion (Focus Group Participant 18).*

*You have to put yourself in other people’s shoes to know how, what it feels like to be there. That is the way you learn compassion (Focus Group Participant 20).*

*You’ve got to listen and you’ve got to know when to talk. You’ve got to know that silence is okay sometimes, because they’re not right with you then… I think listening is the most important thing (Interview Participant 13).*

### Category: sustaining practices: self-care and communities of compassion

While the classroom and clinical setting provided the foundation for training compassion, participants emphasized the necessity of developing personal and professional practices to sustain and enhance compassion across the curricula and over the course of their healthcare career. Participants believed that each healthcare provider was responsible for nurturing compassion, however participants felt that the practice setting and larger system were instrumental to sustaining and enhancing an individual’s ability to provide compassion. In fact, many participants felt that without a practice culture that was conducive to compassion, focusing exclusively on enhancing individual learners’ capacity for compassion would be short-lived and even futile. There were two themes that emerged from the data within this category: Self-Care; and The Need and Responsibility of Practice Settings and Healthcare Organizations in Sustaining and Further Developing Compassion Training.

#### Theme: self-care

Most participants noted that learners should be taught what constitutes compassion, and concurrently, how to sustain themselves through self-care. Many provided anecdotal examples about the impact that occupational stress, burnout and personal stressors had on their caregiving, including their ability to provide compassion, emphasizing the need for training on self-care and educating healthcare providers about additional resources to support them.

*If you don’t care for yourself you can’t give them compassion ‘cause you’re out, you’re empty’ (Focus Group Participant 29).*

*Maybe there’s something and changes that you need to make within that so that you can continue to have compassion. Finding an outlet, whether, sometimes for people it’s exercise, for other people it’s going to the movies. I believe everyone should be in therapy to [laughter] have someone safe like that that you can process and talk to. All of that I think is important (Focus Group Participant 20).*

#### Theme: communities of compassion: the need and responsibility of practice settings and healthcare organizations in sustaining and further developing compassion training

Participants were clear that there was a significant, and often unrecognized, role that the healthcare system played in compassion. The assumption that learners can be trained to provide compassion in a clinical setting was felt to be predicated on a practice environment that values, imparts and embeds compassion in clinical training and as an organizational priority. Participants felt that this responsibility extended from the point of hire to ongoing learning opportunities, as without such a supportive environment, the individual efforts of healthcare providers and compassion training programs were thought to be ineffective. Participants felt that, in addition to training, if compassion was to be considered a core competency, then practice settings and healthcare organizations needed to make compassion an organizational expectation and build in tangible practices and design spaces to support this.

*You know from a system perspective you know I think if you’ve got a work environment, a team, you know your colleagues, your manager and up the chain of command all the way through the system… I also think that there’s relational factors you know with that other individual (Interview Participant 8).*

*Just having that cultural expectation in the culture of the workplace would go a long way. Because then there would be some understanding amongst all the providers about how to support one another and providing that kind of care (Interview Participant 9).*

*My instinct is to leave a situation, I can’t be helpful, I don’t know what to do, but when you have those opportunities to debrief and they say, no, you did this right, or this is what works for me. Sometimes I try seeing it this way. I think you can absolutely, like [name] was saying and [name] was saying, help sort of teach those skills and help cultivate (Focus Group Participant 11).*

## Discussion

In this study participants agreed that compassionate behaviours could be taught but were less certain about how to develop training to cultivate the underlying virtues and affective and relational components of compassion. In part, this was due to a recognition that compassion training was dependent on the innate qualities, life experience and interpersonal skills that individuals possess prior to training. In relation to the HCP compassion model (Fig. 1), this suggests that the themes of virtues, presence and intent within the Virtuous Intent domain, which function as motivators of compassion and distinguish it from routine care, are best cultivated through contemplative practices—whether in terms of a personal practice or by reflecting intentionally on HCPs clinical practice. As such, participants in this study felt that while baseline compassion aptitude varies, learners can enhance the innate qualities and the affective and relational components of compassion through three areas of training: self-awareness, experiential learning and effective and affective communication skills.

Action aimed at alleviating the suffering of an individual is a central and distinguishing aspect of compassion in comparison to sympathy or empathy [33]. In recognizing the inherent challenges in training aimed at cultivating the requisite qualities and affective and relational domains of compassion, study participants suggested that one way learners can cultivate the internal virtues associated with compassion is by providing them with opportunities to engage in compassionate action within their clinical training and practice. Accordingly, while changing learner attitudes can lead to behavioural change, recent research within the field of social psychology suggests that the opportunity to integrate them into practice is not only imperative to sustaining attitudinal change but may change the innate qualities of learners in the process [34–36]. Exposing learners to clinical case scenarios or experiences requiring compassionate action illustrated within the categories and themes within the relational space domain of the HCP compassion model, may in turn function as a positive feedback loop that develops the innate qualities within the virtuous intent domain—cultivating virtues through action. In support of this, functional neural plasticity studies show that compassion training using techniques based on Eastern contemplative traditions activates neuro regions in the brain associated with the positive affective states of reward, love, affiliation and concern for another [37]. Finally, these findings on HCPs perspectives of compassion training and the multidimensional nature of compassion as illustrated in the compassion model (Fig. 1) suggest that compassion training needs to focus on equipping learners with the behaviours, skills, knowledge and qualities spanning the entirety of the HCP Compassion Model and not simply a single dimension, which while developing the virtues associated with compassion through meditation, for example, is void of behavioural or clinical communication training.

Although participants provided many specific suggestions for compassion training based on their own education and practice experience, they were unified in their view that compassion training is currently lacking and needed in healthcare education—affirming the findings of previous studies [1, 6]. Some research has attempted to identify the specific skills or experience that would fill this training gap [38]. One report revealed that the actions that underlie the provision of compassion should be grounded in an understanding of a patient’s values, beliefs and needs [38]. This suggests that teaching compassion using simple statements such as “do what is right” limits a learner’s understanding of what is required of a compassionate care provider, and that compassion mandates caring for patients as they would wish to be cared for, not as a provider would wish to be cared for [38]. A previous study investigating patients’ recommendations suggested that compassion training should focus on developing a connection with the patient and seeing the patient as a person; patient-centered communication, reflective practice and role modeling were identified as important for compassion training [5].

Compassion training programs have emerged considerably in recent years [39]. One review identified eight compassion-based training programs, six of which have been investigated in randomized clinical trials. These include Compassion-Focused Therapy [40], Mindful Self-Compassion [41], Compassion Cultivation Training [26], Cognitively Based Compassion Training [42], Cultivating Emotional Balance [43], and Compassion and Loving-Kindness Meditations [44]. All these training programs include meditation interventions that intend to engender compassion to others and self. A key limitation of these training programs is that none of the approaches have been evaluated in healthcare providers and their patients, despite this being an essential primary outcome of any compassion training program to be used within the healthcare setting. Furthermore, they are all based on differing definitions of compassion and target a variety of competencies [39]. A second review of interventions for compassionate nursing care identified 25 interventions based on staff training, staff support or introducing a new care model to practice [45]. However, none of the interventions were fully described or rigorously investigated. Several interventions evaluated patient-based outcomes, including patient anxiety and satisfaction, but only showed a moderate effect [46, 47]. These findings demonstrate that compassion training is currently inadequate and emphasize that the quality, effectiveness and comprehensiveness of compassion training programs could benefit from an empirical foundation that identifies the core domains of compassion that training programs need to address and a validated measure to evaluate how these educational opportunities impact learner and patient outcomes.

Most participants in the current study identified formal structured learning modules that are experiential, and incorporate the patient perspective as potential learning mediums. Digital narratives of compassionate clinical interactions have previously been used to effectively elicit reflective thinking and discussion with nursing students [27], suggesting this as a feasible approach to compassion training. The importance of a mentoring relationship in training learners to become compassionate HCPs was also emphasized. In addition to role-modeling compassion in practice, participants indicated that mentors are essential for debriefing and serving as an emotional buffer that allows healthcare students to engage in compassion while they explore how best to sustain themselves in the process. Studies in healthcare fields have found that mentoring improved medical student satisfaction with their training programs, increased mentee knowledge and skills, enhanced mentee personal and professional development and improved organizational outcomes [5, 48, 49]. Currently however, clinical mentors are an overlooked educator of compassion, who need to be supported and trained in compassion as they impart their ‘applied’ knowledge of compassion to learners.

Study participants felt that sustaining compassion training required HCPs to practice self-care. Accordingly, personal and professional self-care is increasingly accepted as critical in sustaining the satisfaction and longevity of HCPs [50–53]. Personal self-care recognizes that an individual’s inner-life, family, work, community and spirituality contribute to their personal and professional life [50, 54] and involves strategies that include tending to close relationships such as those with family; following a healthy lifestyle (regular sleep, exercise, vacations); prioritizing time for recreational activities and hobbies; contemplative practices; and engaging in spiritual development [50, 55–57]. Professional self-care strategies may be individual or team-based. Individual professional self-care strategies involve regular evaluation of work life; establishing a network of peers and mentors; improving communication and management skills; setting limits; and reflective writing [50, 58]. Team self-care strategies recognize that team structure and the team processes contribute to a team’s well-being and include empowering team members to empathize with others; developing formal structures, policies and procedures to guide team meetings; and sharing personal and professional opinions on meaning [50, 59, 60]. Self-awareness is a vital aspect of self-care. In HCPs, self-awareness has been defined as the ability to combine self-knowledge with an understanding of the needs of the patient [50]. Self-awareness among HCPs has been reported to result in higher job engagement, better self-care and improved quality of patient care and patient satisfaction [50].

Self-care in HCPs is especially important because compassion specifically requires a willingness on the part of HCPs to be professionally and personally vulnerable by ‘suffering with’ their patients. A previous report found that nursing students had concerns about their own emotional vulnerability when learning to provide compassion [61], while a separate study reported that medical residents felt they needed to limit their compassion in order to meet the demands of their clinical training [62]. Likewise, nursing students managed their fears in part by choosing when to engage in and when to move away from providing compassion, based on the perceived impact on their own emotional health [61]. While self-care is an important facilitator of compassion, placing the onus of responsibility for enhancing compassion on individual HCP efforts, especially since most suggested self-care activities occur outside of their professional lives, was felt to be short-sighed by study participants and failed to account for the responsibilities of the culture of care wherein HCPs practiced.

As a result, study participants felt that support from healthcare faculties and healthcare organizations was essential for sustaining compassion training. However, the dissonance caused by training systems that recognize and value the provision of compassion and “real-world” systems where compassion is often usurped by economics, efficiencies and practice targets [1, 63–65], has been identified by medical and nursing students as a significant theory-practice gap in care [64, 66]. The current study suggests that healthcare education institutions and the organizations that healthcare students and providers work in need to partner in order to address this gap, alleviating the dissonance within HCPs and ensuring that learnings in the classroom are congruent with practice. While the current study and published literature have focused on training HCPs, this novel finding emphasizing the instrumental role that healthcare organizations and practice settings play in sustaining healthcare providers’ training, suggests that compassion training targeted toward healthcare administrators, clinical managers, preceptors and policy makers may be as important as clinical training. The characteristics of a supportive environment include leaders who act as positive role models, positive interdisciplinary relationships, autonomy in the workplace, compassion toward colleagues and an intentional focus on staff well-being [24, 67, 68].

The current study is limited in sampling only palliative care providers. However, almost all clinicians who participated in this study have extensive clinical experience beyond provision of palliative care. Future research is needed to replicate the results of this study in other fields of healthcare. Further, there may have been sample and selection bias. Our use of convenience, snowball and theoretical sampling meant study participants sentiments about the importance and content of compassion training was reflective of individuals who were self-motivated to participate and had an interest in the topic. Because interviewees were nominated by their peers as exemplary compassionate care providers, they may have only nominated individuals whose perspectives were congruent with their own and there may be some element of selection bias on the part of interviewees in the responses provided. Finally, we conducted the focus groups and interviews within a Western, well-resourced, publicly funded, team-based healthcare system and therefore findings may not be generalizable to the provision of compassion in other cultures where educational opportunities and healthcare practices vary.

## Conclusions

Providing compassion has become a core expectation of healthcare and increasingly is being considered a core competency of healthcare education. Providing evidence based, clinically informed training to help learners practice with compassion is crucial to their success, to organizational outcomes, and most importantly, to providing quality patient care. Based on the results of this study, we suggest that a combination of formal experiential learning modules and clinical mentorship that focuses on teaching learners techniques to engage with persons, self-awareness, and practices for sustaining the ability to provide compassion are necessary for training compassionate care providers. In order to achieve this, further research is needed to determine learner needs, along with systematic reviews and environmental scans of existing training to determine needs, content, teaching methods and the barriers and facilitators of existing training programs. Healthcare organizations are instrumental to provision of compassionate health care delivery, as the success and longevity of training clinical teams or individual HCPs in compassion will be contingent on the degree that organizations cultivate sustain, and consider a culture of compassion as a core business of healthcare.

## Data Availability

The datasets used and/or analyzed during the current study are available from the corresponding author on reasonable request.
